# Portable and label-free optical detection of sweat glucose using functionalized plasmonic nanopillar array

**DOI:** 10.1038/s41378-025-01152-6

**Published:** 2026-01-26

**Authors:** Ling Liu, Kuo Zhan, Joni Kilpijärvi, Matti Kinnunen, Yingqi Zhao, Yuan Zhang, Mulusew W. Yaltaye, Yang Li, Artem Zhyvolozhnyi, Anatoliy Samoylenko, Seppo Vainio, Jianan Huang

**Affiliations:** 1 Research Unit of Health Science and Technology, Faculty of Medicine, University of Oulu, 90220 Oulu, Finland; 2 Biocenter Oulu, 90220 Oulu, Finland; 3 Polar Electro Oy, Kempele, Finland; 4 Faculty of Biochemistry and Molecular Medicine, Disease Networks Research Unit, University of Oulu, FI-90014 Oulu, Finland; 5 InfoTech Oulu, Kvantum Institute University of Oulu, FI-90014 Oulu, Finland

**Keywords:** Nanophotonics and plasmonics, Biosensors

## Abstract

Continuous glucose monitoring (CGM) is vital for diabetes care, but current invasive electrochemical sensors of blood glucose often cause potential infection and skin irritation. Non-invasive sensors in sweat glucose are promising alternatives but limited by low sensitivity and poor compatibility with complex sweat environments, because the sweat glucose has concentrations of 20 – 600 μmol/L and are 100-fold more dilute than the blood glucose. Here, we report a portable optical sensing system that integrates an optical watch prototype with functionalized plasmonic silver-coated silicon nanopillars substrate for non-invasive and label-free glucose detection in sweat. The nanopillar sensor with wide-range plasmonic hot spots is functionalized with 4-mercaptophenylboronic acid for selective glucose capture and optical signal transduction through both Raman scattering and plasmonic detection. The optical watch system has a compact LED illumination at 623–660 nm and wireless transmission of data to a smartphone application. Significantly, the whole system demonstrated excellent sensitivity down to 22 μmol/L and high selectivity in detecting glucose in artificial sweat, which were validated by human sweat samples to confirm its applicability in real-life scenarios. Our study offers a promising portable and non-invasive alternative to traditional CGM and highlights the potential of integrating nanophotonic sensors with wearable platforms for continuous health monitoring and personalized medicine.

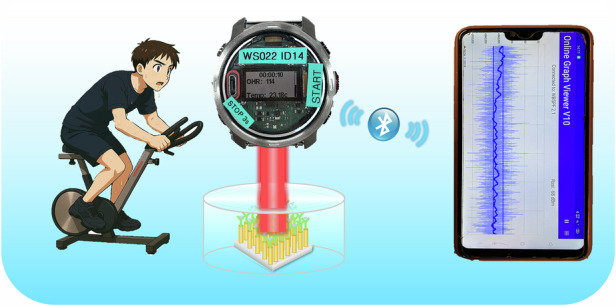

## Introduction

Diabetes mellitus affects over 537 million individuals worldwide and remains one of the most prevalent chronic diseases and leading causes of mortality^1–3^. Effective management of the disease requires frequent monitoring of blood glucose levels to avoid complications and maintain metabolic balance^4,5^. Although continuous glucose monitoring (CGM) technologies have significantly advanced diabetes care, most existing systems rely on subcutaneous electrochemical sensors^6^. These minimally invasive approaches often result in discomfort, increased risk of infection, and reduced patient compliance, particularly during long-term use. Consequently, there is an increasing need for a non-invasive, reliable, and user-friendly glucose monitoring strategy suitable for prolonged daily applications.

Sweat, an easily accessible and information-rich body fluid, has emerged as a promising candidate for non-invasive biomarker monitoring^7–9^. It contains various physiologically relevant analytes, including glucose, electrolytes, and lactate, and its composition correlates with blood chemistry under specific conditions^10–12^. The presence of abundant sweat glands throughout the human skin enables straightforward and continuous sample collection^13,14^. However, although the concentration of glucose in sweat is correlated with blood glucose levels^15,16^, it is typically 10 to 100 times lower than that in blood and shows significant variability. Glucose concentration in the sweat of healthy individuals is typically only 20–600 μmol/L^4,15^, making it technologically challenging to achieve adequate detection sensitivity and specificity. Furthermore, interference from other sweat components, erratic secretion rates and the complex environment on the skin surface make it more difficult to reliably measure sweat glucose concentration^17–19^.

Several sensing strategies have been investigated to address these obstacles. Electrochemical sensors are commonly employed due to their compact form and rapid response^20^. However, they may cause irritation to the skin and exhibit poor selectivity in complex biological matrices. Optical methods such as colorimetry^21–23^ and fluorescence^24^ offer improved sensitivity. But they typically depend on bulky components and intricate optical systems, including excitation light sources and detectors. This in turn restricts their integration and application into wearable devices. In addition, Lideberg and Nylander who first demonstrated the use of surface plasmon resonance (SPR) in optical biosensors in 1982^25,26^. Due to the specific binding sites in and ability to detect biomolecular interactions in real time without labeling^26–29^. The SPR technology has been adapted to such fields as biology, health sciences, drug development, clinical diagnosis, and environmental and agricultural monitoring. However, the SPR based fiber optic sensor solution challenges such as high detection limits and insufficient response sensitivity. The devices have been hard to miniaturize, fluid stability need to be optimized an sensitivity still increased for low concentration analytes to achieve better disease diagnostics potential^30,31^, For these reasons, integration or SPR with wearable platforms remains a key technological development issue^32,33^.

**Fig. 1:**
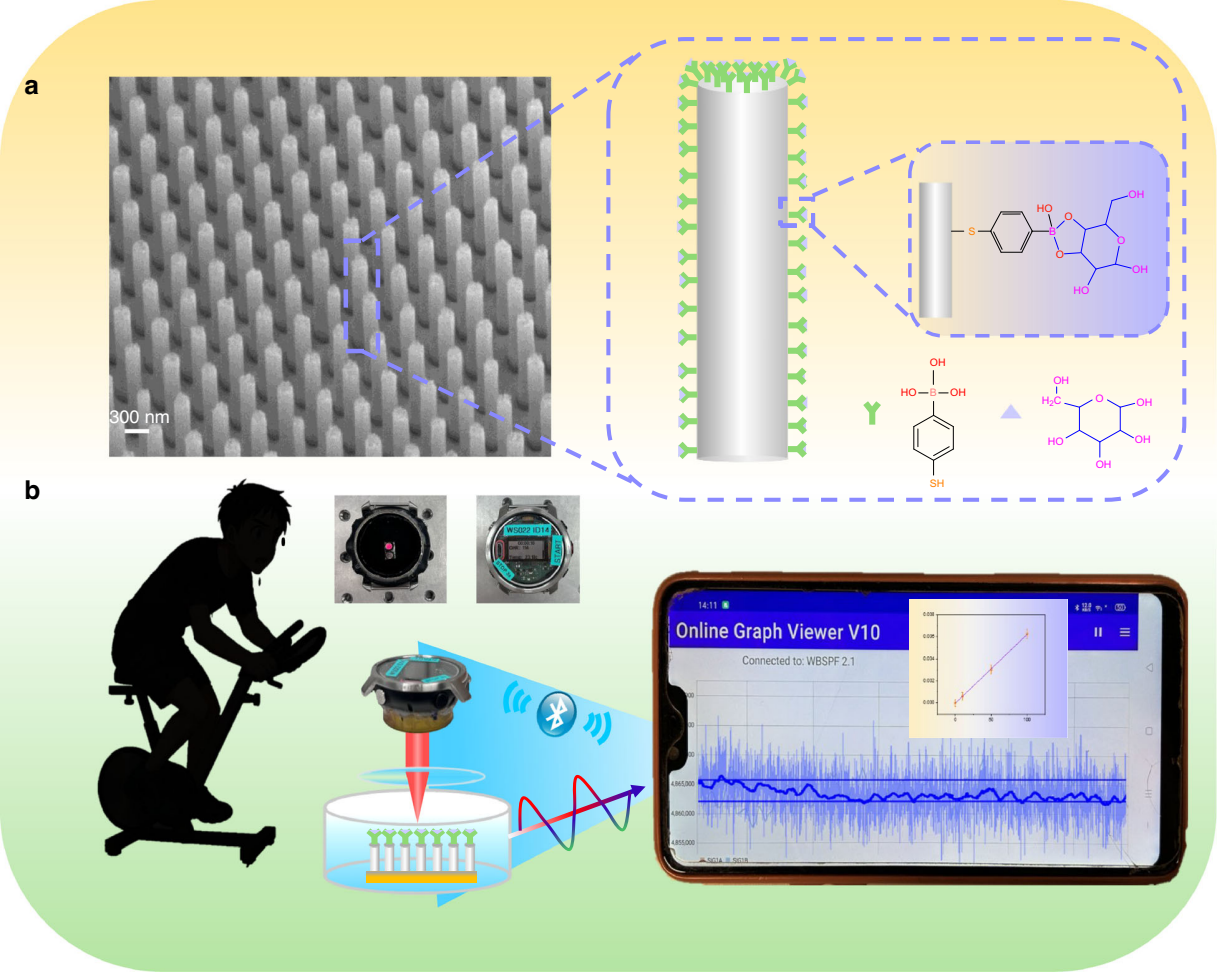
The schematic diagram shows a glucose detection platform that integrates a plasma silver nanopillar sensor with an optical watch system. **a** Schematic and SEM image of the plasmonic silver nanopillars functionalized with 4-MPBA for selective bind glucose. **b** Schematic illustration of the optical watch prototype system for non-invasive glucose monitoring and real-time data synchronization

In this work, we developed a portable plasmonic sensor for measuring sweat glucose by combining a plasmonic nanopillar substrate with an optical watch prototype (Fig. 1). The plasmonic nanopillar structure exhibits a strong localized surface plasmonic resonance (LSPR) with wide-range plasmonic hot spots^34^ upon simple LED illumination that supports both surface-enhanced Raman scattering (SERS) and LSPR-based plasmonic detection. The nanopillars were functionalized with 4-mercaptophenyl-boronic acid (4-MPBA) as selective glucose receptors, enabling transduction of binding events into optical signals through changes in the local refractive index. Before plasmonic detection, the SERS signals of the 4-MPBA and glucose binding were used to confirm binding of glucose on the nanopillars.

We developed the potential wearable sensors and optimized their sensitivities of glucose with instruments from Raman microscope, portable Raman spectrometer, portable fiber spectrometer to optical watch prototype. A compact LED with wavelengths of 623–660 nm and a photodiode detector are integrated into an optical watch prototype system and provides wireless data transmission to a smart phone application for non-invasive detection as shown in Fig. 1b. The developed device enables accurate quantification of glucose in artificial sweat within physiologically relevant ranges, achieving a detection limit as low as 22 μmol/L. The feasibility and reliability of the portable nanopillar sensor for label-free, non-invasive glucose detection were further confirmed by using sweat samples from human volunteers and comparing them with an enzyme kit as a reference method. This work presents a significant advance toward the realization of wearable photonic devices for real-time health monitoring and offers new opportunities for personalized diabetes care.

## Results

### Sensitivity and specificity of glucose binding

Accurate detection of glucose in sweat remains a fundamental challenge due to its low concentration and the presence of numerous interfering substances^4^. Unlike blood, where glucose levels are relatively stable and abundant, sweat contains 20–600 μmol/L^4,15^ of glucose that fluctuates also based on hydration and physical activity. Furthermore, the Raman scattering cross section of glucose molecules is 5.6 × 10⁻³⁰ cm²·molecule⁻¹·sr⁻¹, which is about 14 orders of magnitude lower than that of typical fluorescent molecules^34–36^. This makes its Raman signal extremely weak. In the presence of complex biological background noise, signal acquisition becomes particularly difficult^37^. To solve the problem of the weak Raman signal of glucose molecules, we first constructed a plasmonic gold nanopillar structure. This structure exhibits good array uniformity and structural stability (fabrication details in Materials and Methods and Fig. S1). Under visible light excitation, it produces a strong localized surface plasmon resonance (LSPR) effect, forming a highly localized electromagnetic “hot spots” on its surface and significantly enhancing the Raman scattering signal of target molecules adsorbed on its surface. To achieve highly selective glucose detection in a complex sweat environment, we functionalized the gold nanopillars with 4-mercaptophenylboronic acid (4-MPBA), firmly anchoring the 4-MPBA to the gold surface via Au-S bonds. The boronic acid group in the 4-MPBA molecule binds specifically to the cis-diol structure on the glucose molecule. This significantly enhances the probe’s affinity for glucose and its molecular recognition ability^37,38^. This structural and molecular recognition strategy creates an optically active interface on the gold nanopillar surface, significantly amplifying the weak glucose Raman signal via dual SERS and LSPR mechanisms. We used scanning electron microscopy (SEM) and surface-enhanced Raman spectroscopy (SERS) to systematically evaluate the structure, morphology, and stability of the 4-MPBA-functionalized plasmonic gold nanocylinders. Figure S2 shows that the nanocylinders’ morphology remains largely unchanged at room temperature for up to 30 days. Figure S3 shows that the SERS signal remains stable throughout the entire testing period. Together, the substrate exhibits excellent long-term structural and performance stability, providing strong support for its practical application in sweat glucose detection.

**Fig. 2:**
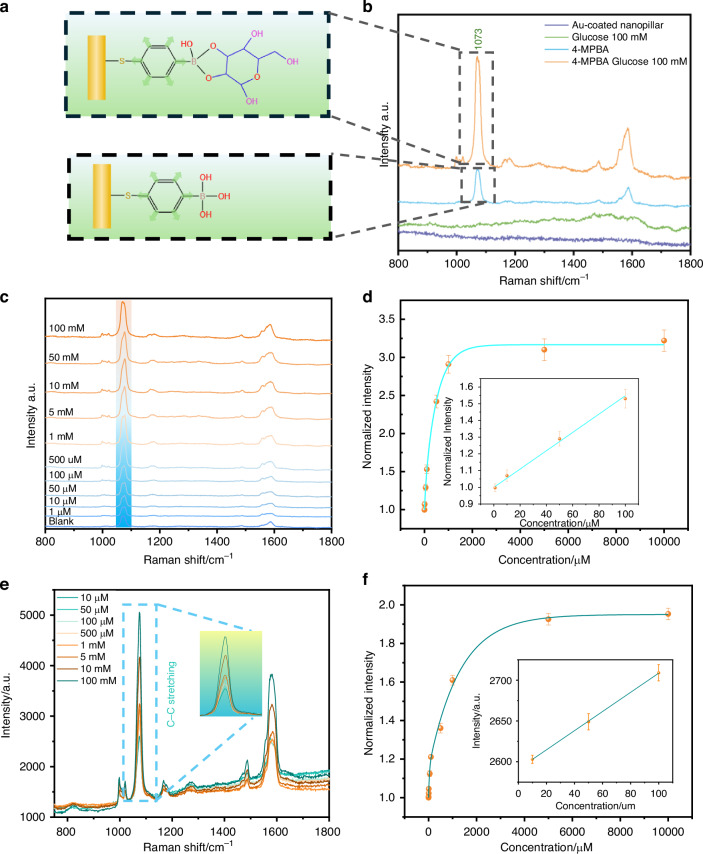
SERS performance of the plasmonic nanopillars. **a** Surface structure and glucose binding of 4-MPBA-functionalized gold nanopillar. **b** SERS spectra of gold nanopillar (purple line), 100 mM glucose solution (green line), functionalized gold nanopillar (blue line), and functionalized gold nanopillar in glucose solution (orange line) using the confocal Raman microscope. **c** SERS spectra of 4-MPBA-functionalized Au nanopillars versus glucose concentrations by the confocal Raman microscope. **d** Calibration curve of normalized SERS intensity of 4-MPBA functionalized gold-coated nanorods versus glucose concentration at the 1073 cm^-1^ peak from (**c**) with LoD of 117.9 μmol/L. **e** Portable Raman spectra of 4-MPBA functionalized Au nanopillar versus glucose concentration. **f** Calibration curve of normalized SERS intensity of 4-MPBA functionalized gold-coated nanorods versus glucose concentration at the 1073 cm^-1^ peak from (**e**) with LoD of 224.3 μmol/L

Our results demonstrate that the 4-MPBA-modified nanopillars generated a distinct SERS peak at 1073 cm⁻¹ corresponding to the C-C stretching of the aromatic ring, which exhibited a significant intensity increase in the presence of glucose^37^. As shown in Fig. 2a, b, the comparison between bare gold nanopillars, 4-MPBA-functionalized gold nanopillars, and glucose-exposed samples clearly confirms the molecular recognition capability and enhancement effect of the sensing interface. As shown in Fig. 2c, the SERS signals of glucose solutions with different concentration gradients (0-10,000 μmol/L) were detected using confocal Raman microscopy. Extracting the intensity of the characteristic peak at 1073 cm⁻¹ and normalizing it showed that the SERS signal intensity increased with glucose concentration. In the 0-100 μmol/L range, the signal exhibited a strong linear correlation with concentration (fitted equation: y = 1.00602 + 5.3557x; slope: 5.3557; R² = 0.991), with a limit of detection (LoD) of 117.9 μmol/L. To validate and optimize the detection strategy further, a portable Raman spectrometer detected glucose at the same concentration gradient (Fig.2e). The results are shown in the Fig. 2f. A good linear response was also observed in the 0–100 μmol/L range (fitted equation: y = 1.00019 + 4.5717x, slope = 4.5717, R² = 0.999), corresponding to an LoD of 224.3 μmol/L. These findings indicate that the combination of molecular recognition via 4-MPBA and nanostructure-assisted optical enhancement effectively overcomes the long-standing barrier of selectivity and sensitivity in sweat-based glucose sensing. This molecular interface serves as a robust platform for reliable detection in biologically relevant concentration ranges, laying the foundation for practical non-invasive applications.

### Material optimization for portable plasmonic detection

**Fig. 3:**
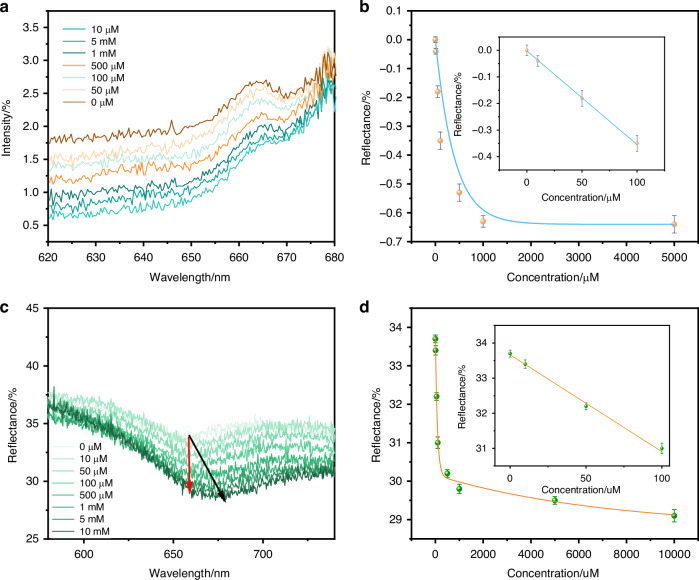
Portable plasmonic detection of glucose with a portable fiber spectrometer. **a** The relationship between the LSPR reflectance intensity of functionalized gold-coated nanopillars and the glucose concentration by a portable LSPR spectrometer. **b** Relationship between the reflection intensity and glucose concentration under the action of 4-MPBA modified gold nanopillars sensor achieved LoD of 330 μmol/L. **c** The relationship between the LSPR reflectance intensity of functionalized silver-coated nanopillars and the glucose concentration by a portable LSPR spectrometer. **d** Relationship between the reflection intensity and glucose concentration under the action of 4-MPBA modified silver nanopillars sensor LoD of 20.9 μmol/L

To apply the nanopillars to potential wearable optical devices, we firstly use a portable fiber spectrometer to excite the Localized surface plasmon resonance (LSPR) of the gold nanopillars by simple backscatter illumination (Fig. S4) against traditional SPR configurations relying on bulky gold thin films on prism-based optical setups^39^. Figure 3a shows the results of the LSPR technique for various glucose concentrations. The reflectance spectrum is located at approximately 670 nm, and the reflectance intensity gradually decreases with increasing glucose concentration. In the low concentration range of 0–100 μmol/L, a good linear relationship was observed between change in reflectance intensity (ΔReflectance Intensity) and glucose concentration. The fitted equation was y = -0.0024–0.00352x, with a slope of -0.00352. The goodness of fit R² = 0.998, as shown in Fig. 3b. The corresponding limit of detection (LoD) was 330 μmol/L. However, the LoD of gold nanorods cannot meet the concentration range of glucose in human sweat^4,15^. To address this limitation, we explored material substitutes by replacing the gold layer with a silver coating on the silicon nanopillar substrate. Compared to gold, silver exhibits sharper and more intense plasmonic enhancement in the visible and red regions due to its lower imaginary permittivity and higher plasmonic quality factor. Scanning electron microscopy (SEM) images (Fig. S5) show that the silver nanopillars have a high aspect ratio and a uniform surface structure, which effectively enhanced the LSPR effect, thereby improving the detection sensitivity of glucose molecules.

Measurements using a portable fiber optics spectrometer confirmed that the resonance clarity and sensitivity were significantly improved when using the silver nanopillars substrate. As shown in Fig. 3c, the reflectance valleys showed distinct red shifts and decreasing intensities in response to increasing glucose concentration. The silver-based substrate exhibited a clear, good linear response to glucose within the low micromolar concentration range (0–100 μM). The fitting results showed that the response curve followed the linear equation y = -0.0335–0.01003x, with a slope of -0.01003 and an R² = 0.994 indicating a good fit. As shown in Fig. 3d, the detection limit (LoD) of this system can reach 20.9 μmol/L, which is nearly an order of magnitude more sensitive than previous results. To ensure accuracy in frequent daily testing for diabetes patients, the disposable silver nanocolumn substrate used in this study effectively avoids signal interference caused by repeated use. Under short-term testing conditions, chlorides in sweat have almost no effect on the signal (Figs. S6 and S7), further ensuring the stability and reliability of the test. This material optimization strategy not only enhances detection performance but also facilitates the design of wearable systems: the sensor can be directly integrated with compact, low-cost, low-power red laser diodes for operation. By intensity changes within this spectral window, the optical and practical application requirements of miniaturized, battery-powered platforms can be met.

### System integration and measurement of the optical watch prototype

**Fig. 4:**
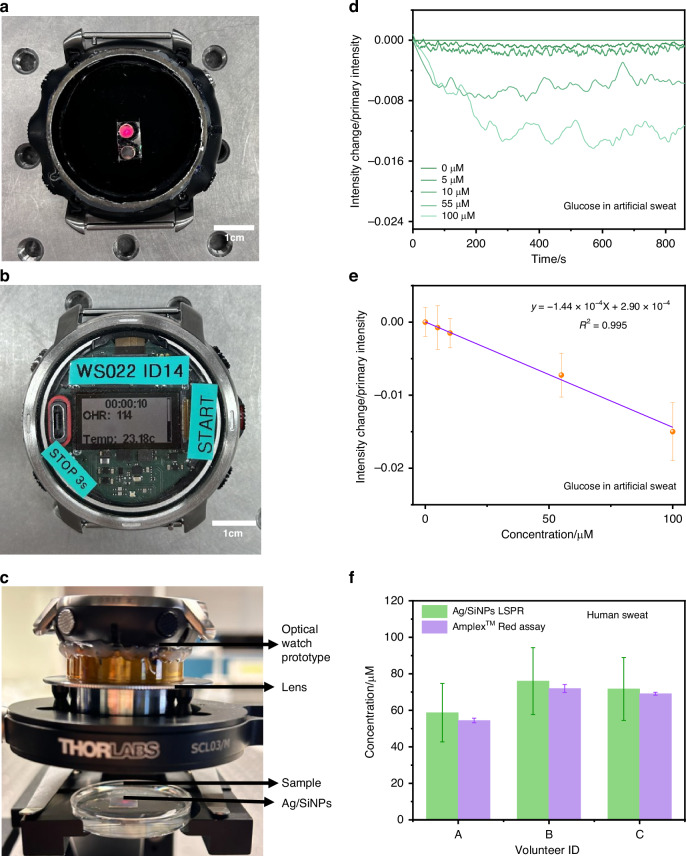
Wearable plasmonic detection with an optical watch prototype. **a** the back of the optical watch prototype where the red dot is the LED and the one next to it is the photodiode. **b** The front of the optical watch prototype that indicate the wireless communication circuit and operational screen. **c** The measurement setup. **d** The normalized curve of reflected light intensity change relative to initial intensity shows the time-dependent sensing response of the Optical watch prototype to glucose solutions in artificial sweat of various concentrations (0–100 μmol/L). **e** Linear relationship between glucose concentration and intensity change/primary change (ΔI/I), with a LoD of 22 μmol/L. **f** Test results of 3 healthy volunteer sweat samples using Ag/SiNPs LSPR and Amplex™ Red Glucose Assay

Although many optical sensing technologies provide high analytical performance in controlled laboratory settings, they are limited in their ability to be translated into practical, real-time applications due to device complexity, power consumption, and a lack of user-friendly interfaces. To address these challenges, we developed an optical watch prototype - a fully integrated, potentially wearable system that combines a compact LED (623–660 nm), a photodiode, and a wireless Bluetooth communication module connected to a smartphone app (Figs. 4a, b). A commercial Polar optical watch that uses the same LED has been tested according to relevant technical standards and has been found to pose no risk to users. Its performance parameters are ~ 5 mW power consumption, a 22 Hz sampling rate, and a ~ 1 day battery life. During measurements, the photodiode continuously records the intensity of reflected light and transmits it to the app. We chose the wavelength based on the previously studied LSPR performance of silver nanopillars to achieve the strongest plasmonic response while maintaining low power consumption. In addition, by adding a lens between the watch and the nanopillar chip, the light focusing effect and signal collection efficiency are further enhanced (Fig. 4c). With this apparatus, we can capture signal variations in real time during the sweat detection process. This lays a crucial foundation for applying optical sensing technology to potential wearable diagnostic devices.

### Validation using human sweat samples

Due to the composition of human sweat is complex and may interfere with the results detected by the sensing system, we first used artificial sweat containing Na^+^/K^+^, Ca^2+^, lactic acid, urea and etc., to verify the reliability and feasibility of the sensor. We created standard glucose concentrations in artificial sweat solutions (Fig. 4d) and plotted a standard curve (Fig. 4e) based on the relative intensity change/primary change (ΔI/I) over the 0 to 100 μmol/L concentration range. The fitting equation was y = −1.44 × 10^−4^x + 2.90 × 10^−4^, R² = 0.995. Based on these results, we calculated a limit of detection of 22 μmol/L for artificial sweat samples. This range covers glucose concentrations found in the sweat of healthy individuals and diabetic patients, demonstrating the sensor’s potential for daily blood glucose management in nonclinical settings. Furthermore, we prepared a 30 μmol/L glucose solution in the artificial sweat and spiked them in the artificial sweat with glucose concentrations of 10, 30, and 50 μmol/L for testing, as shown in Fig. S8. We analyzed the obtained result using the standard curve model established for artificial sweat to predict spiked glucose concentrations, as shown in Table S1. By comparing the predicted and spiked concentrations, the recovery rates were calculated according to literature^40^, The recovery rates fall within the range of 80–120%. These results demonstrate that the sensor maintains excellent selectivity and accuracy under complex matrix conditions, exhibits minimal interference effects, and achieves satisfactory recovery rates.

To evaluate the applicability of this wearable system in real-world scenarios, we conducted a preliminary study with three healthy volunteers. We used a prototype optical watch to detect sweat samples collected from volunteers while they were cycling, and connected it to a smartphone to view real-time data. As illustrated in Fig. S9, the reflectance spectra of sweat samples from all three volunteers exhibited a downward trend over time. The results were analyzed by combining the changes in relative intensity/primary changes (ΔI/I). The glucose concentration in sweat samples from three volunteers was then predicted using an established artificial sweat standard curve model. To verify the reliability of the results, we used the Amplex™ Red Glucose/Glucose Oxidase Assay Kit as a reference standard^41^. First, the same series of artificial sweat of different glucose concentrations were tested by the Amplex™ Red Glucose Assay Kit as shown Fig. S10a. A linear curve was obtained based on concentration and fluorescence intensity (Fig. S10b). It is important to note that the Amplex™ Red Glucose Assay is based on a glucose oxidase-peroxidase coupled reaction. The enzymic kit’s detection relies on the fluorescence signal of the product, thus requiring a fluorescence detection instrument. In contrast, the optical watch prototype provides direct signal output, eliminating the need for external instrumentation. We tested sweat samples from three volunteers using the Amplex™ Red Glucose Assay and predicted the glucose concentration in human sweat from the calibration curves. Figure 4f shows the detected sweat glucose concentrations of the three volunteers and demonstrates the high detection accuracy of the nanopillar sensors. This result shows the feasibility and potential application of the optical watch in real sweat detection. The detected glucose concentrations in human sweat by the nanopillar sensors (58.7 ± 16, 76.0 ± 18.3, 71.7 ± 17.2 μmol/L) were a bit higher and have larger standard deviation than those from the enzymic kit (54.4 ± 1.3, 71.9 ± 2.2, 69.1 ± 0.8 μmol/L) for all 3 volunteers. The difference in detected concentrations might come from the different detection mechanism: while nanopillar sensors detected the glucose bond on the pillar surface, the enzymic kit can detect glucose in the whole illuminated volume of sweat. On the other hand, the larger standard deviation may origin from the instruments: the enzymic kit used a benchtop microplate reader to obtain stable signals, the optical watch prototype for the nanopillar sensors exhibited limited signals stability. However, the method of this study is still in the preliminary stage of clinical validation. Due to the limited sample size, it is not yet sufficient to fully reflect its applicability in clinical practice. In the future, we will further expand the clinical sample size and continue to optimize the detection method and sensor design to improve its stability and universality.

## Discussion

The system improved light collection efficiency by using a lens between the optical watch and the culture dish. This lens effectively focuses the LED light on the nanopillar chip and guides the signal to the photodiode inside the watch. However, since sweat samples must still be collected manually, the system cannot dynamically monitor patients’ blood glucose levels while being worn, which limits its practicality. To truly realize wearable sweat sensors, our future work will improve the optical watch prototype as a lab-in-a-watch system by integrating within the watch: 1) six photodiodes surrounding the LED; 2) iontophoretic sweat stimulation electronics; and 3) optofluidic module that includes the lens, a slot for fixing the nanopillar chip at the focal plan of the lens and a microfluidic channel to direct the stimulated sweat to the chip.

Taking together, the results presented in this study demonstrate a comprehensive strategy for overcoming key limitations in non-invasive and label-free glucose detection. By integrating a functionalized nanopillar substrate with an optical watch prototype, our system achieves high sensitivity and selectivity for glucose detection in complex sweat environments. The successful replacement of gold with silver nanopillars in the LSPR substrate significantly improved signal response in the red-light region, which is critical for low-power wearable applications. In addition, the real-time wireless data transmission supported by the optical watch system provides a user-friendly interface for personalized health tracking, which allows users to intuitively view blood glucose changes without relying on external devices, thus bridging the gap between high-performance biochemical sensing and user-centered health technologies. Compared with existing portable optical sensors in Table 1, our work demonstrated high sensitivity of glucose in human sweat with a low detection limit as 22 μM.

**Table 1:** Comparison of the performance of similar research work of portable, label-free optical sensors

Reference	Detection mechanism type	Readout method	Repeatability	LoD (μM)	Linear Range (μM)	Wireless data transmission	Biological samples
**This work**	LSPR	Portable reader	High	22	0-100	Yes	Human sweat
**Ranta-Lassila, A. et al**.^30^	SPR	Portable reader	Middle	5890	0-56000	No	Artificial solution
**DUC LE et al**.^33^	SPR	Portable reader	High	14200	0-28000	No	Artificial solution
**Daejong Yang et al**.^37^	SERS	Bench-top Raman instruments	High	NA	100-30 000	No	Aqueous humor in rabbit eyes.
**Kien Voon Kong et al**.^44^	SERS	Bench-top Raman instruments	middle	100	100-10 000	No	Urine samples
**Koh et al**.^21^	Colorimetric sensing	Smartphone	High	~200	NA	Yes	Human sweat
**Chunhua Lin et al**.^45^	Colorimetric sensing	UV-Vis absorbance	High	2.8	10-1000	No	Serum samples

Unlike many existing wearable sensors that rely on electrochemical principles and suffer from limited selectivity or skin irritation, our optical approach provides a label-free, minimally intrusive solution with strong potential for long-term use. The dual-mode sensing capability (SERS and LSPR), combined with molecularly selective functionalization via 4-MPBA, establishes a robust platform adaptable to other biomarkers beyond glucose. Future work will focus on expanding the sample size for clinical validation, improving the stability of the sensor under long-term use, and integrating advanced data analysis algorithms for intelligent health feedback. The modularity of this sensing system also opens the door to multiplexed detection and broader applications in wearable diagnostics.

## Conclusion

This study presents a concept wearable surface plasmon resonance (LSPR) sensor system based on functionalized silver nanopillars. This system is designed for the non-invasive, label-free detect of glucose content in human sweat. The platform uses 4-mercaptophenylboronic acid (4-MPBA) as a selective molecular recognition interface combined with nanostructure-enhanced optical sensitivity to achieve a detection limit of 22 μmol/L for artificial sweat. The Amplex™ Red Glucose Assay has validated the accuracy and reliability of portable optical sensors for detecting glucose in 3 volunteer sweat. This demonstrates that the sensors can maintain stable performance within physiologically relevant concentration ranges. These results demonstrate the potential of combining nanoplasmonic materials, molecular recognition chemistry, and micro-optics to develop practical, wearable biosensors. The proposed system is a promising approach for noninvasive, user-friendly glucose monitoring and paves the way for the future development of multiplexed wearable diagnostic devices for personalized medicine.

## Materials and methods

### Material

The silicon wafer was Boron-doped p-type with a resistivity of 5–20 Ω·cm^-2^, D-(+)-Glucose and 4-mercaptophenylboronic acid (4-MPBA) were purchased from Sigma-Aldrich Co., LLC. Polystyrene nanospheres of 500 nm in diameter and Amplex™ Red Glucose/Glucose Oxidase Assay Kit (Cat. No A22189) was brought from ThermoFisher Scientific, Artificial human sweat (Cat. No. BZ319) was purchased from Biochemazone Inc (Edmonton, Canada). The composition of artificial sweat is Sodium Phosphate Dibasic, Histidine, Sodium Chloride, Lactic Acid, Acetic Acid, Urea, Calcium Chloride, Ammonium Chloride, Sodium Sulfate, Ammonium Sulfate, Amino acids, Magnesium Chloride Potassium Chloride.

### Fabrication of 4-MPBA-coated plasmonic nanopillars

The silicon nanopillars (SiNPs) array was fabricated based on a previous research^42^. Briefly, polystyrene nanospheres with a diameter of 500 nm and a mixed solution of ethanol and ethylene glycol in a volume ratio of 1:2:1 was slowly injected onto the water surface to form a monolayer. A clean silicon wafer, previously immersed, was slowly raised to the water surface so that the polystyrene monolayer was on top of it. After air drying, the as-made substrate was placed in a reactive ion machine (Plasmalab80Plus RIE) and the polystyrene balls were etched for 3 minutes and 15 s at 30 m Torr pressure, 30 W oxygen power and 5 W argon power. Then, a 2 nm titanium layer and a 5 nm gold layer were then deposited on the substrate using a Q150T ES Sputtering Coating Machine. The substrate was placed in a mixed solution of ethanol and pure water at a volume ratio of 1:1 and ultrasonicated for 5 min. Finally, the SiNPs array was prepared by immersing the substrate in an etchant consisting of 20 mL of water, 1 ml of 48% HF and 0.2 ml of 35% H_2_O_2_ for 10 min.

For the gold- or silver-coated silicon nanopillar arrays (Au/SiNPs, Ag/SiNPs): A Sputtering Coating Machine was used to deposit a layer of gold or silver with a thickness of about 50 nm on the prepared SiNPs array. Finally, the prepared nanopillar array was placed in a 6 mM 4-MPBA solution at room temperature overnight.

### Sweat collection

Ethical permission (EETTMK:110/2015) was approved by ethical committee of Oulu University Medical School according to the Finnish Medical Research Act (488/1999). 3 healthy male volunteers, aged 25 to 35 years, provided written informed consent. Although sweat must be collected via the skin, the skin might contaminate the samples by harboring microorganisms, accumulating analytes, and shedding cells^41^. So before collecting sweat, the collection site (the upper body, arms, and trunk) was gently rinsed with clean water. Soap and other detergents were also avoided for 24 h prior to sweat collection to minimize interference from exogenous pollutants on sweat components. After drinking 500 mL of purified water, volunteers performed 30 min of indoor cycling on the ProSpinner spinning bike to promote sweat discharge. The sweat samples (20–70 mL) were pretreated with a 40 μm cell filter (Fisherbrand, 22363547) and a 0.8 μm microporous filter (Millipore) to remove cells and particulate impurities^43^.

### SERS measurement

Portable Raman Spectrometer: A 638 nm laser with an output power of 50 mW was coupled to the OceanOptics QPro spectrometer, and a fiber optic probe with a focal length less than 0.5 mm was connected. The output power of the 638 nm laser was set to 5 mW, and the integration time was set to 0.1 s to collect the Raman fingerprint spectrum of the glucose sample.

Renishaw confocal Raman microscope spectrometer : After the InVia^TM^ (Renishaw) confocal Raman microscope spectrometer was fine-focused through the 100× objective lens, the output power of the 785 nm laser was 1 mW, the integration time was 0.1 s, and the functionalized 1 × 1 cm gold nanocolumns were placed in a standard concentration of glucose solution and allowed to stand for 15 min before collecting the Raman fingerprint spectrum of the glucose sample.

### LSPR and optical watch prototype measurement

Portable fiber optics Spectrometer: The experiment utilized a red surface plasmon resonance (LSPR) laser with an output power of 50 mW and an operating wavelength range of 620–680 nm, a fiber optic probe with a ball lens, and an OceanOptics QPro spectrometer to create a measurement system. The 1 × 1 cm functionalized gold or silver nanorod substrate was immersed in a standard glucose solution concentration, and the surface plasmon (LSPR) spectral response was collected continuously within 15 minutes.

Optical watch prototype: LSPR data were collected as optical reflectance on the nanopillar chips using an OceanOptics spectrometer with a halogen white light source that coupled to an optical fiber to illuminate and collect optical signals from pure glucose solutions and sweat samples. To measure the reflectance of the nanopillars in the standard-concentration glucose solutions or sweat, the nanopillar chips were placed in a petri dish under the optical watch prototype. A lens was inserted between the watch and the petri dish to direct the signal into the photodiode of the watch. Functionalized 1 × 1 cm gold or silver nanopillars were placed in standard-concentration glucose solutions or sweat and continuously detected for 15 min.

### Amplex™ red glucose/glucose oxidase assay kit test

Mix 100 μL of the sample with 100 μL of the working solution. Let the mixture stand for 30–60 min. Then, perform fluorescence detection using a Tecan Spark multifunctional microplate reader (Tecan Trading AG, Switzerland). The test parameters are an excitation wavelength of 530 nm with a 10 nm bandwidth, an emission wavelength of 570–620 nm with a 10 nm bandwidth, 15 flashes, and an integration time of 30 s.

### ΔI/I calculation and smoothing parameters

During data processing, the original intensity trajectories I(t) are firstly subjected to background noise deduction and their normalized fractional change is calculated as follows:

$$\Delta {\rm{I}}/{\rm{I}}=\frac{I(t)-I0}{I0}$$

Where the baseline intensity *I*_0_ is defined as the average signal intensity during the first 5 s of analyte injection (sampling rate of 100 Hz for 1000 frames).

For visualisation purposes (Fig. 4d), a Savitzky-Golay filter (MATLAB function sgolayfilt) was used to smooth the signal trajectory. The filter parameters are a 500-point window and a second-order polynomial in the fitting order. The window length was chosen to effectively suppress high-frequency scattering noise (>10 Hz) while preserving slow signal jumps with frequencies below 0.1 Hz.

## Supplementary information

SI- clean 1106

## Data Availability

The data that support the findings of this study are available from the corresponding author upon reasonable request.
