# Supplementary material for: Portable and label-free optical detection of sweat glucose using functionalized plasmonic nanopillar array
Source: Microsyst Nanoeng. 2026 Jan 26;12:43. doi: 10.1038/s41378-025-01152-6 (PMC12835093; doi:10.1038/s41378-025-01152-6)
Supplement: Supplementary file 1 — SI- clean 1106 [file 41378_2025_1152_MOESM1_ESM.docx]

***Supporting Information***

**Portable and Label-free Optical Detection of Sweat Glucoses using Functionalized Plasmonic Nanopillar Array**

Ling Liu^1^, Kuo Zhan^1,4*^, Joni Kilpijärvi^2^, Matti Kinnunen^2^, Yingqi Zhao^1^, Yuan Zhang^1^, Mulusew W. Yaltaye^1,3,4^, Yang Li^1^, Artem Zhyvolozhnyi^3^, Anatoliy Samoylenko^3^, Seppo Vainio^3^, Jianan Huang^1,3,4*^

^1^Research Unit of Health Science and Technology, Faculty of Medicine, University of Oulu, Aapistie 5A, 90220 Oulu, Finland.

^2^Polar Electro Oy, Kempele, Finland.

^3^Faculty of Biochemistry and Molecular Medicine, Disease Networks Research Unit, InfoTech Oulu, Kvantum Institute, University of Oulu, FI-90014 Oulu, Finland.

^4^Biocenter Oulu, Aapistie 5A, 90220 Oulu, Finland.


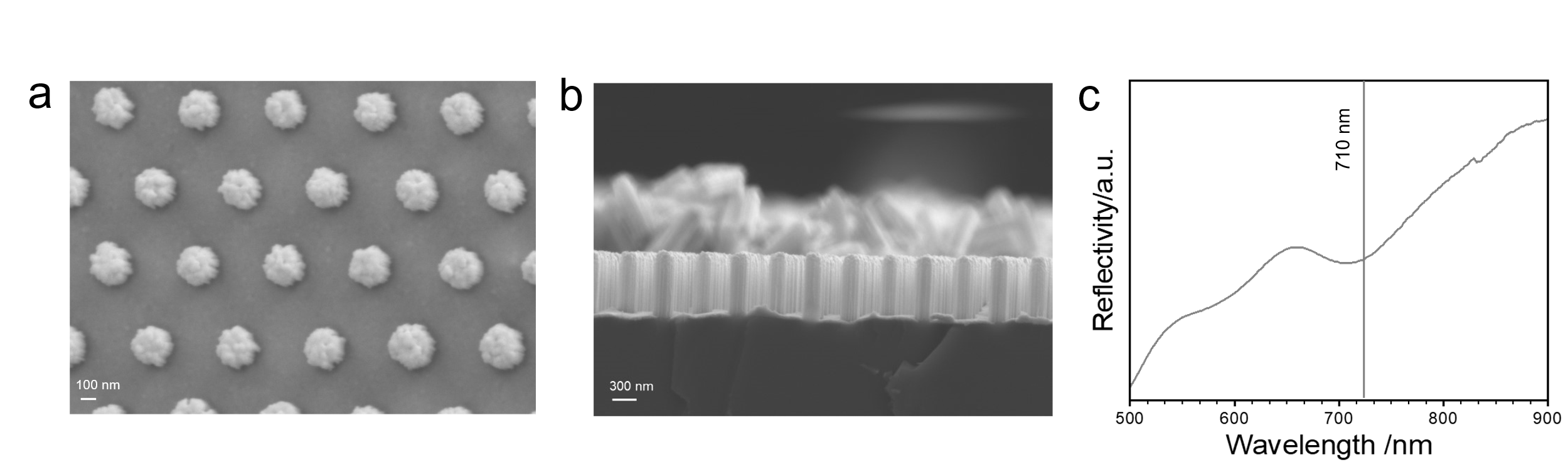


**Fig. S1.**  (a) a top-down SEM image of Au/SiNPs. (b) the SEM image of the cross section of Au/SiNPs. (c) Reflectivity spectrum of Au/SiNPs.


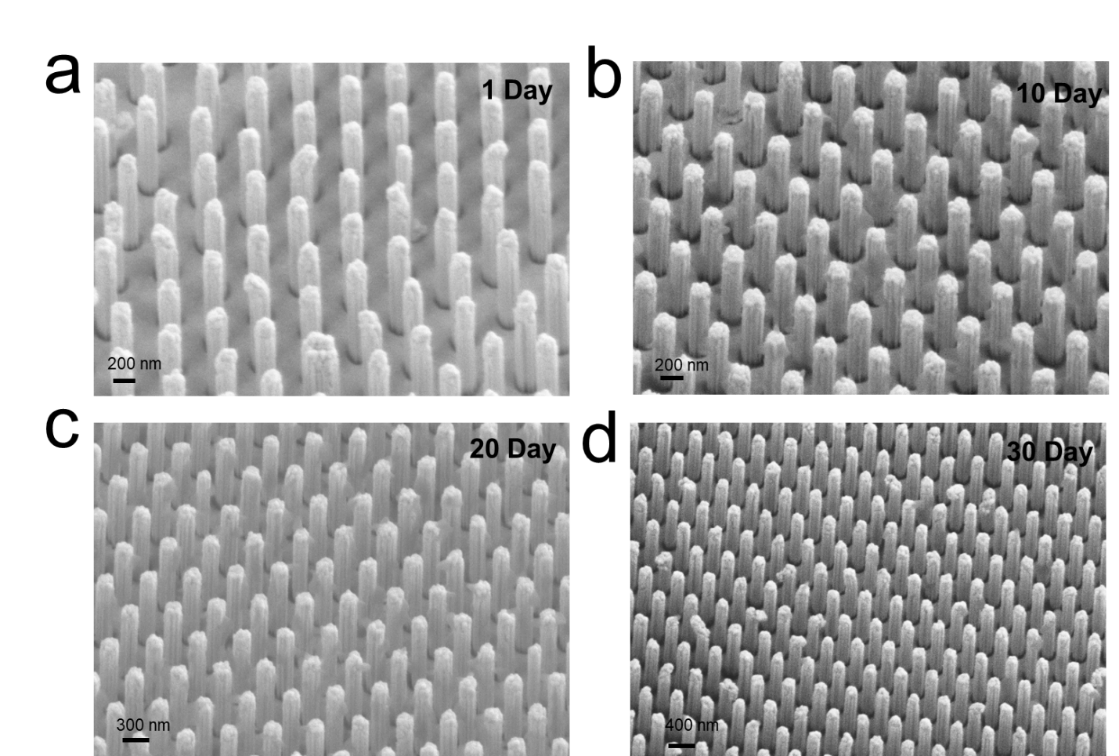


**Fig. S2.** The SEM images of 4-MPBA modified Au/SiNPs substrates at different storage times (1st day, 10th day, 20th day, 30th day) at room temperature.


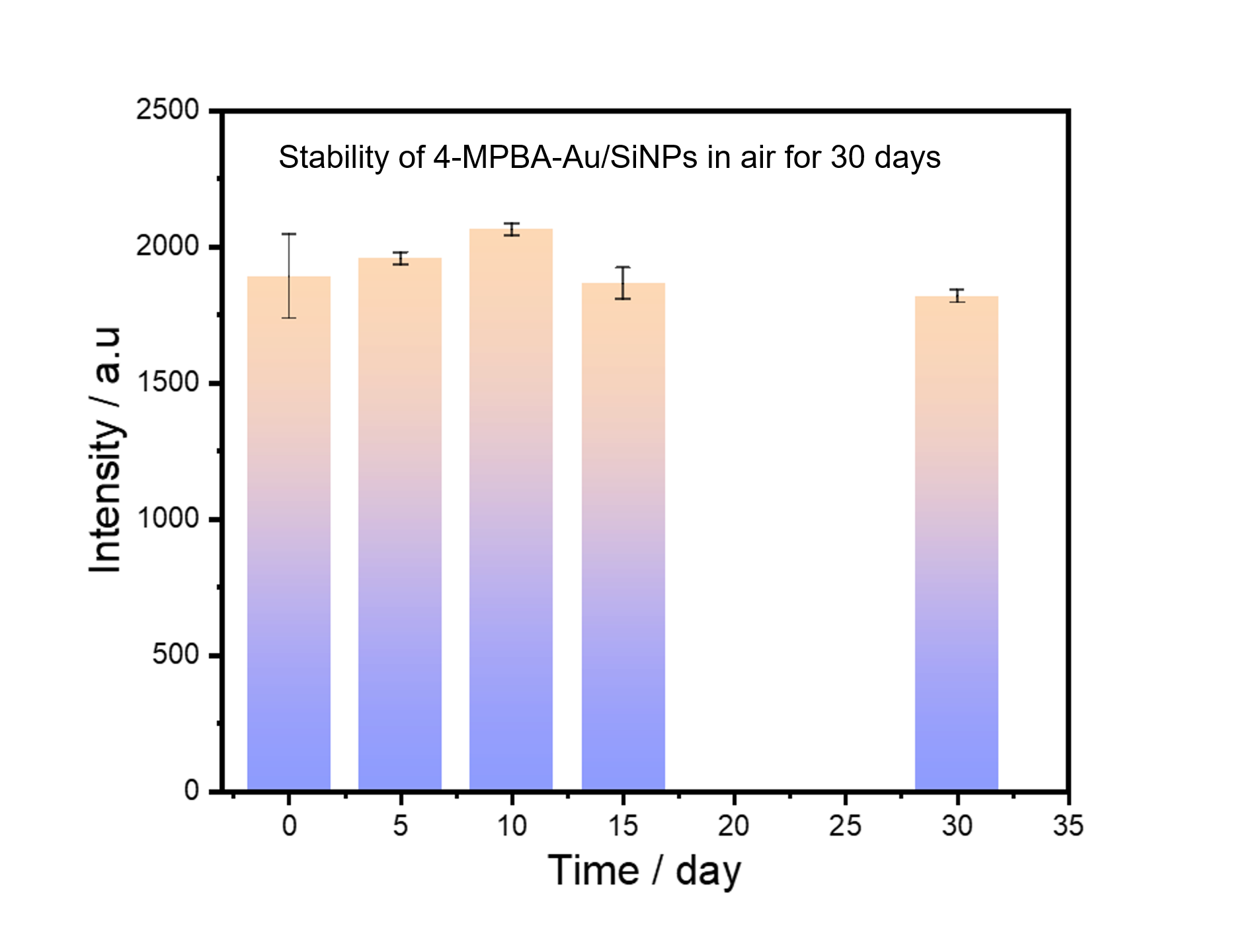


**Fig. S3.** The temporal changes in the Raman intensity of the characteristic peak at 1073 cm⁻¹ when using the 4-MPBA-modified Au/SiNPs substrate to detect 1 mM glucose after storage for different lengths of time at room temperature (20 tests per group).


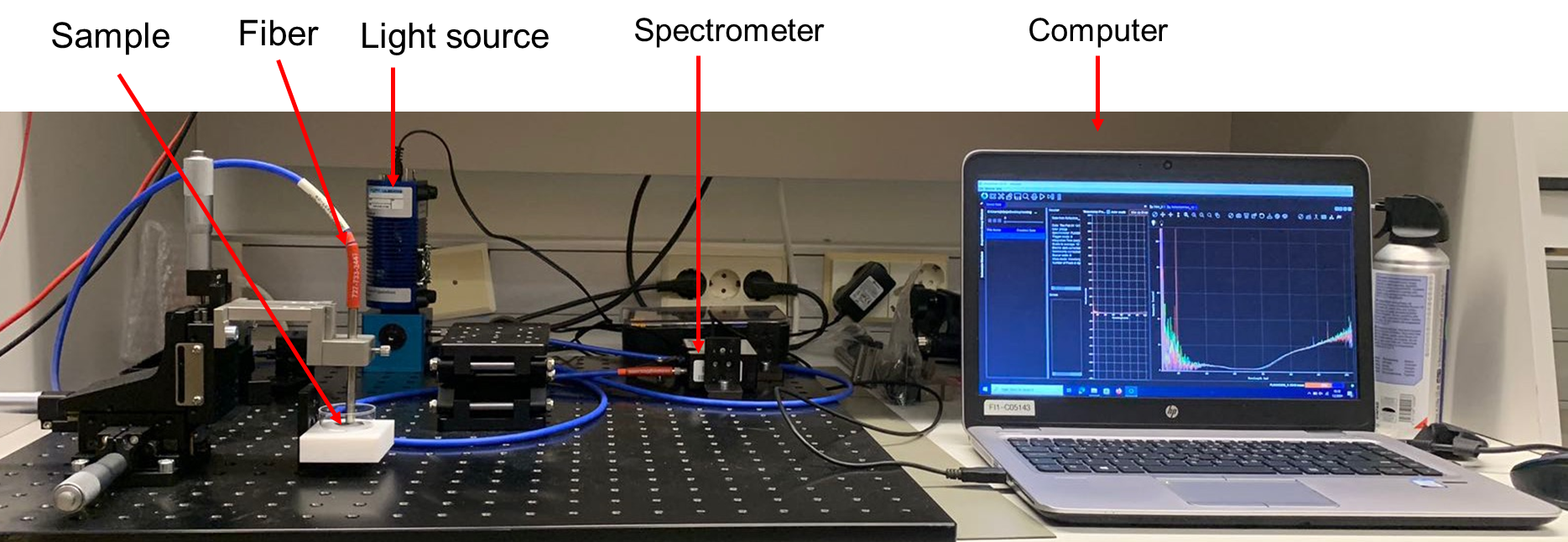


**Fig. S4.** The portable fiber optics spectrometer for localized surface plasmon detection.


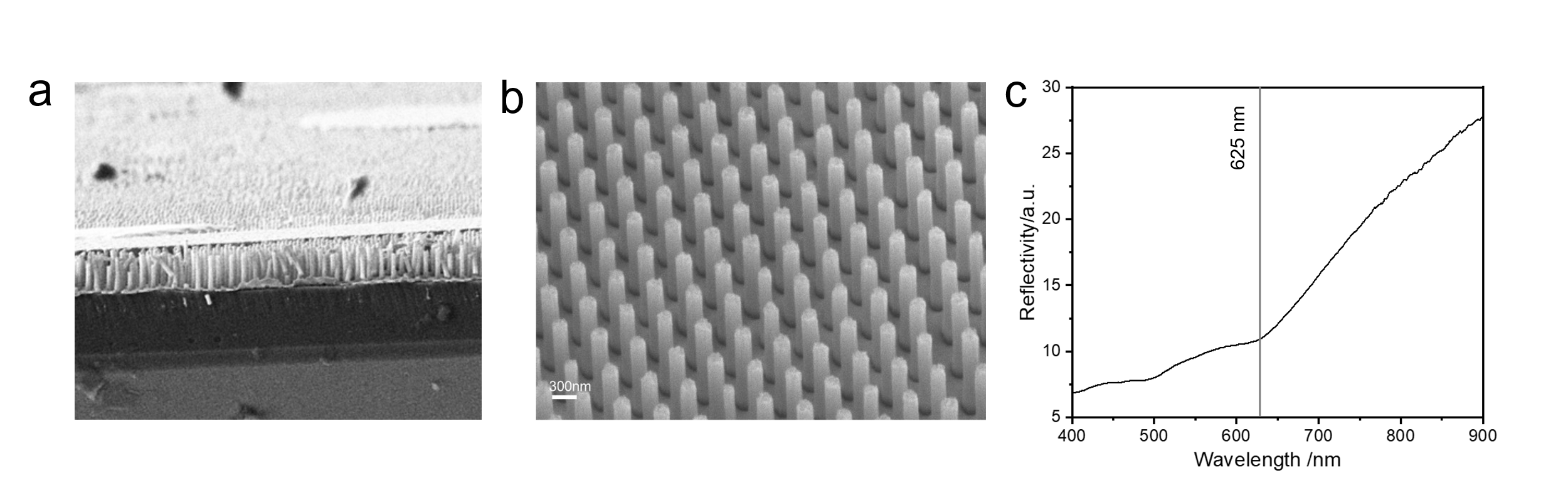


**Fig. S5.** (a) The SEM image of the cross section of Ag/SiNPs. (b) Scanning electron microscope image of Ag/SiNPs taken from top to bottom at a tilt angle of 30°, showing its surface morphology. (b) Reflectivity spectrum of Ag/SiNPs.


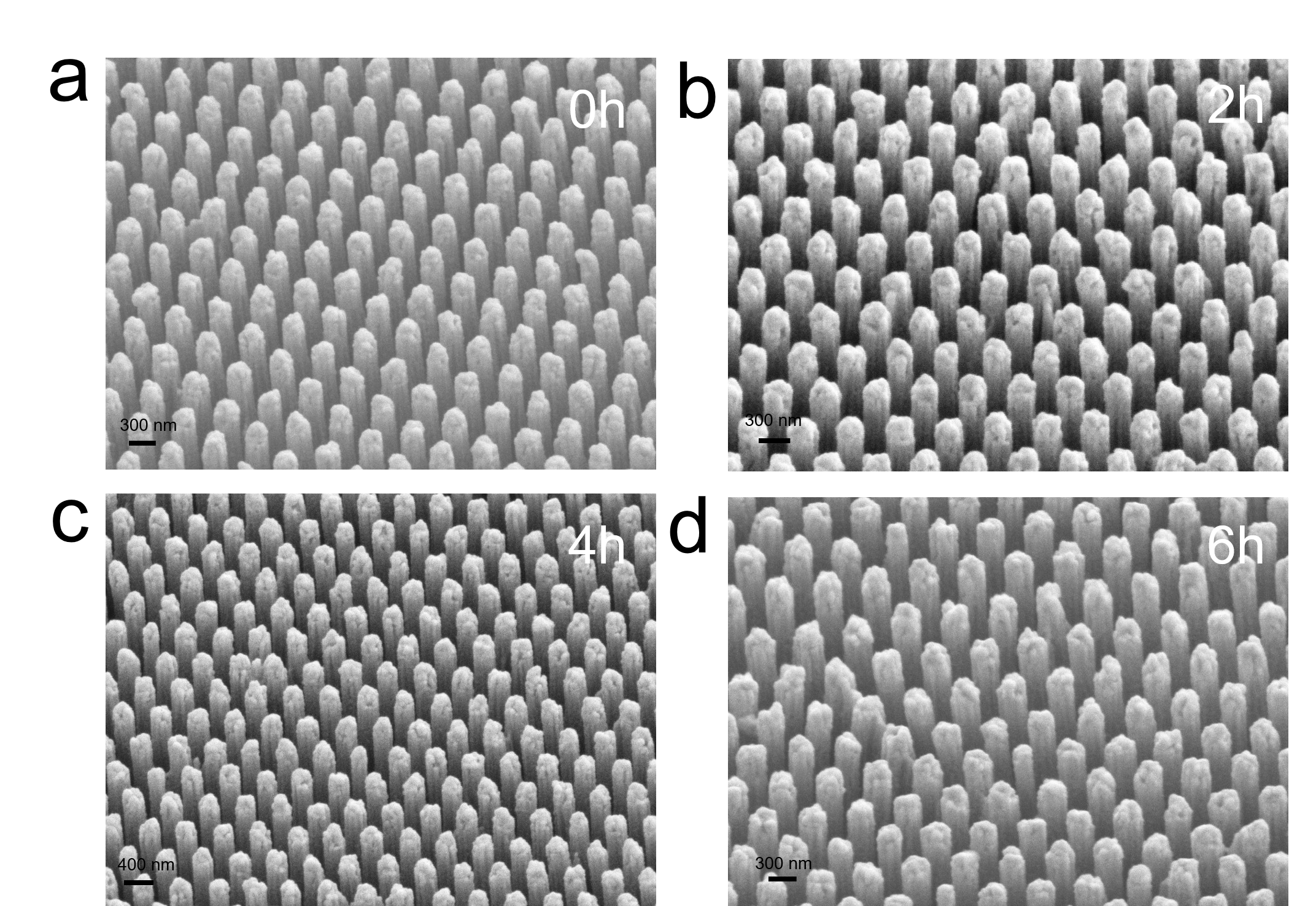


**Fig. S6.** (a) The SEM image of the 4-MPBA-modified Ag/SiNPs immediately after immersion in sweat. (b) The SEM image of the 4-MPBA-modified Ag/SiNPs after immersion in sweat for 2 h. (c) The SEM image of the 4-MPBA-modified Ag/SiNPs after immersion in sweat for 4 h. (d) The SEM image of the 4-MPBA-modified Ag/SiNPs after immersion in sweat for 6 h.


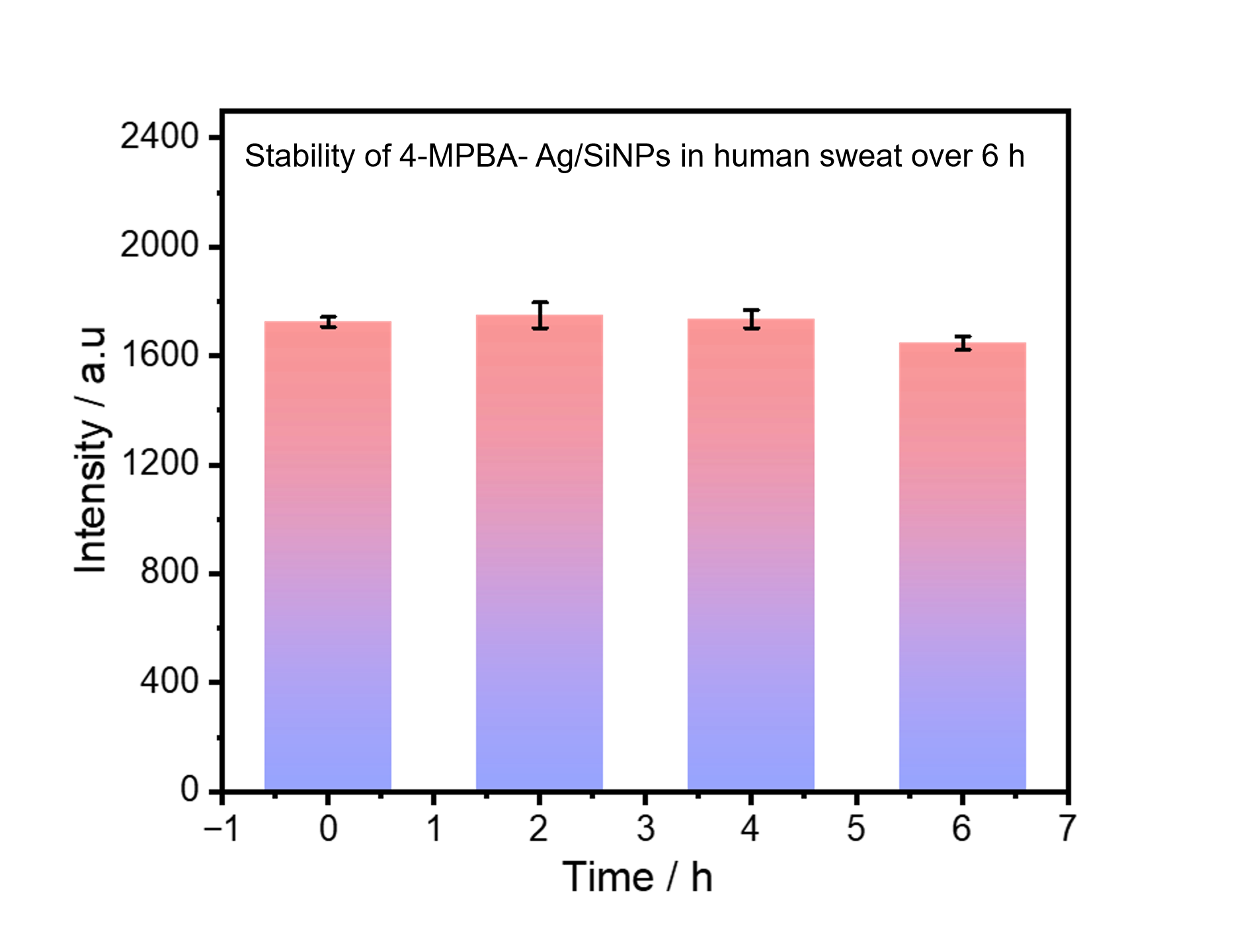


**Fig. S7.** The time-dependent variation in the Raman intensity of the characteristic 1073 cm⁻¹ peak for 4-MPBA-modified Ag/SiNPs during a 6h immersion in sweat from volunteer C.


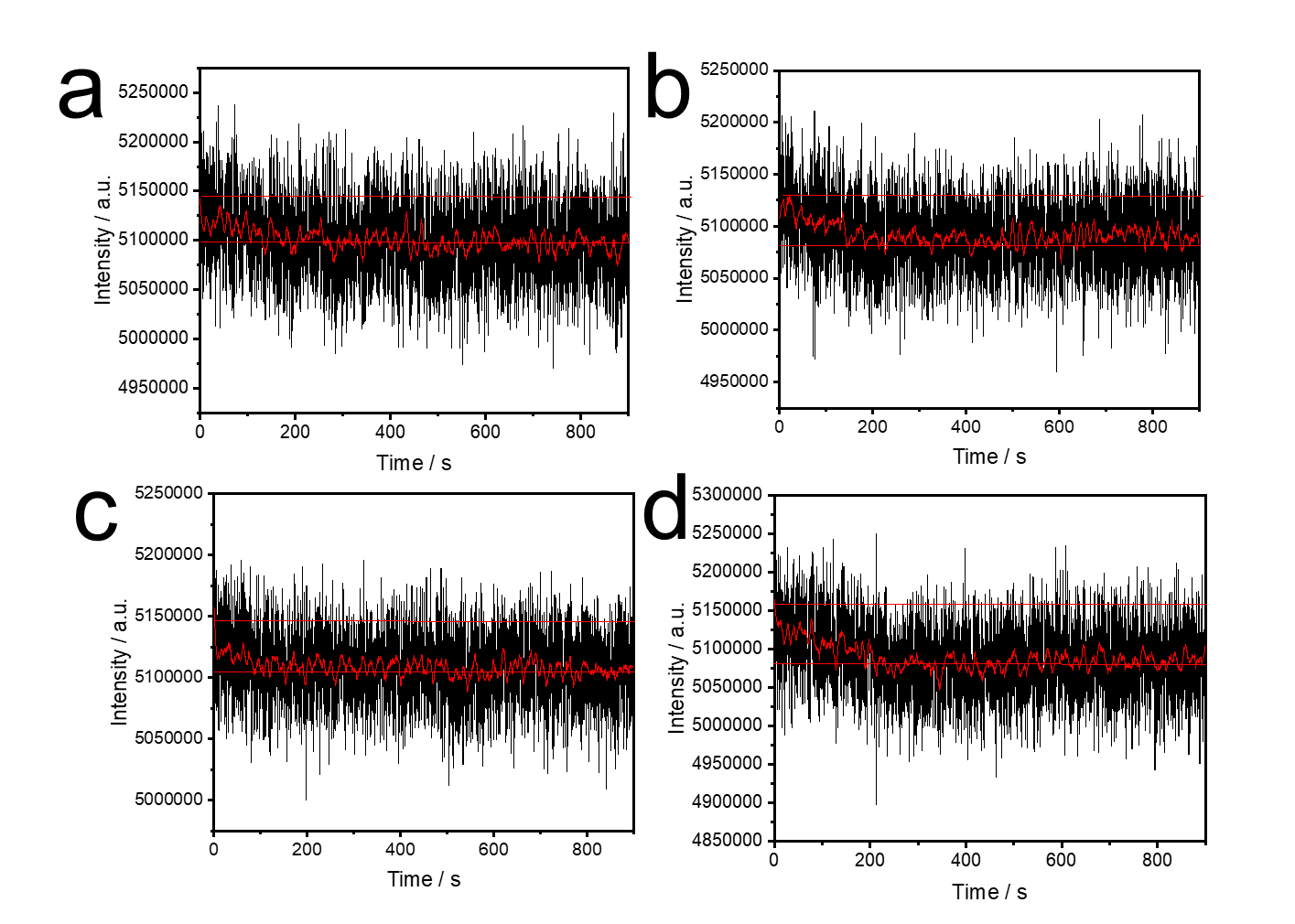


**Fig. S8.** Time domain response characteristics of reflection intensity of (a) 30 μmol/L (b) 40 μmol/L (c) 60 μmol/L (d) 80 μmol/L concentration glucose solution in artificial sweat within a 900 s period.

**Table S1.** Recovery rate results of spiked artificial sweat samples.

| Sample  number | Initial concentration  (μmol/L) | Spiked concentration  (μmol/L) | Actual  Concentration  (μmol/L) | Predicted concentration (μmol/L) | Recovery  rate ^A^  (%) |
| --- | --- | --- | --- | --- | --- |
| 1 | 30 | 10 | 40 | 38.3 | 83% |
| 2 | 30 | 30 | 60 | 57.7 | 92.3% |
| 3 | 30 | 50 | 80 | 84.1 | 108.2% |

^A^Recovery rate = [$\frac{Predicted concentration- Initial concentration}{Spiked concentration}$] × 100.


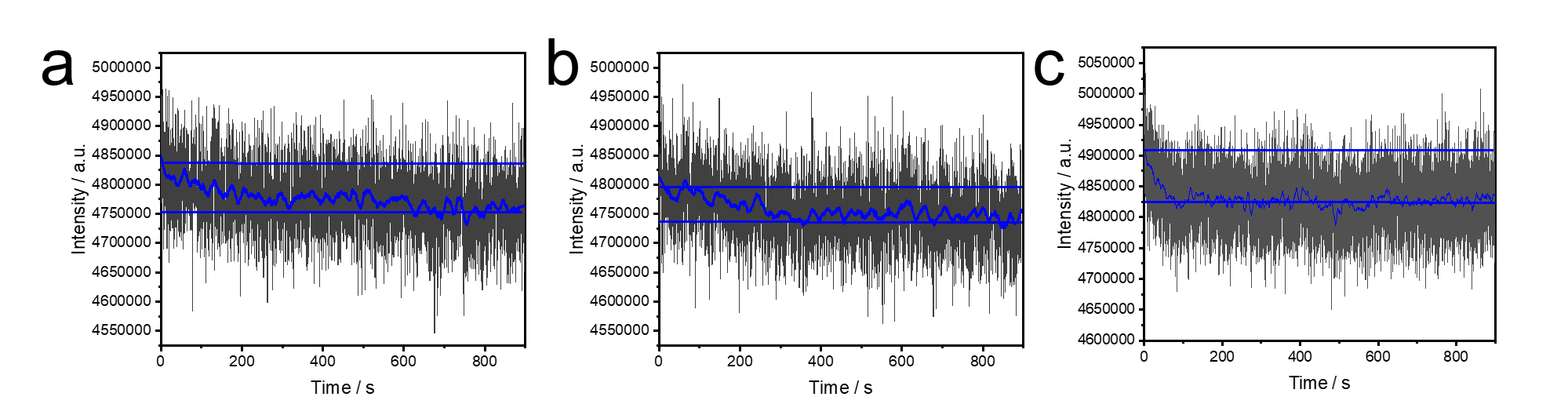


**Fig. S9.** The time domain response characteristics of the reflection intensity of 3 human sweat samples over a period of 900s.


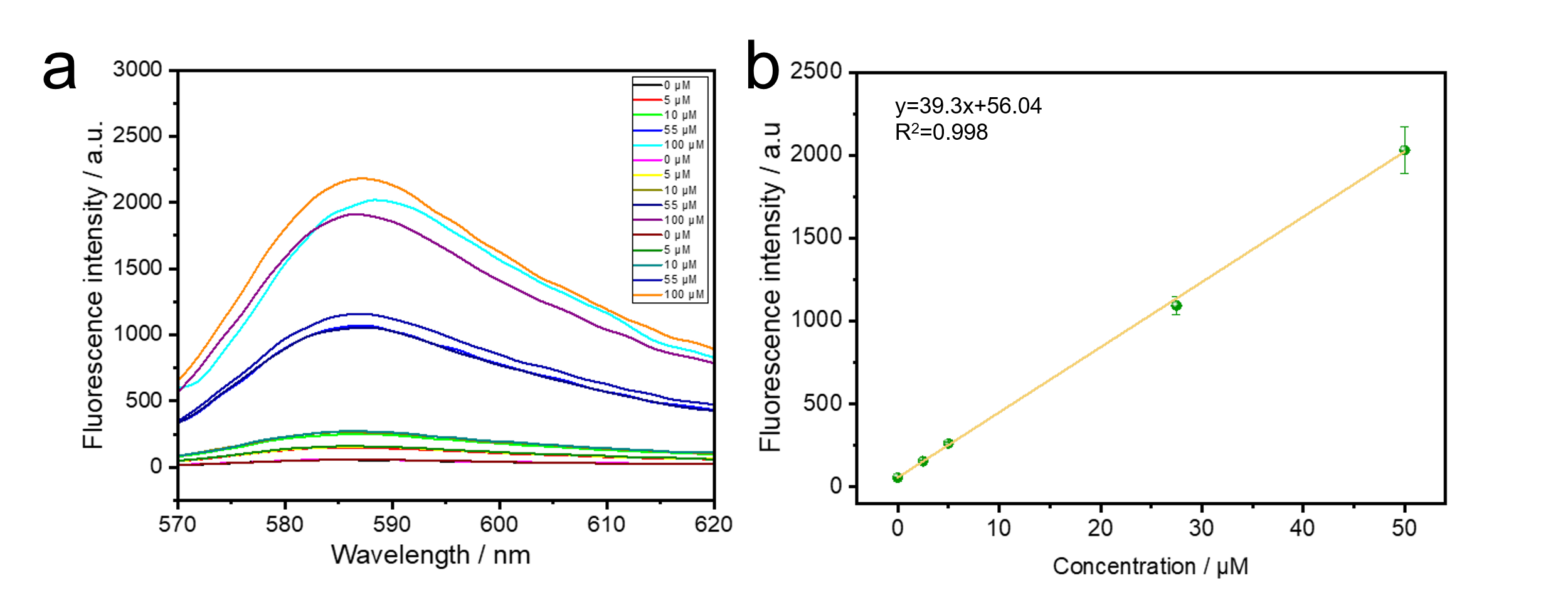


**Fig. S10.** (a) Glucose standard solutions ranging from 0–100 μmol/L were prepared in artificial sweat and their fluorescence intensity was measured. Three independent experiments were performed at each concentration point. (b)The standard calibration curve was drawn based on the different glucose concentrations in artificial sweat and the corresponding fluorescence intensity.
